# Human herpesvirus type 8 in tuberculosis patients with effusion

**DOI:** 10.1186/s12879-015-1179-2

**Published:** 2015-10-30

**Authors:** Shih-Ming Tsao, Chun-Liang Lai, Ming-Nan Lin, Jen-Pi Tsai, Cheng-Chuan Su

**Affiliations:** Institute of Medicine, Chung Shan Medical University, Taichung, Taiwan; Sections of Infectious Diseases and Chest Medicine, Department of Internal Medicine, Chung Shan Medical University Hospital, Taichung, Taiwan; School of Medicine, Tzu Chi University, Hualien, Taiwan; Department of Internal Medicine, Buddhist Dalin Tzu Chi Hospital, Chiayi County, Taiwan; Department of Family Medicine, Buddhist Dalin Tzu Chi Hospital, Chiayi County, Taiwan; Department of Clinical Pathology, Buddhist Dalin Tzu Chi Hospital, 2, Minsheng Road, Dalin Town, Chiayi County, 622 Taiwan; Department of Anatomic Pathology, Buddhist Dalin Tzu Chi Hospital, Chiayi County, Taiwan

**Keywords:** HHV-8, Imunofluorescence assay, Kaposi’s sarcoma, Primary effusion lymphoma, Tuberculosis

## Abstract

**Background:**

Many patients with tuberculosis (TB) are seropositive for human herpesvirus type 8 (HHV-8), and many patients with primary effusion lymphoma have high levels of HHV-8 DNA in their effusions. However, the status of HHV-8 in the effusions of patients with TB remains unclear.

**Methods:**

Blood samples were collected from 129 patients with pulmonary TB and 129 age- and sex-matched healthy controls. Forty of the TB patients had pleural or peritoneal effusions, and 38 of these effusions were available. Both blood and effusion samples were analyzed for lymphocyte and monocyte counts and/or HHV-8 antibodies and DNA.

**Results:**

TB patients with or without effusions had significantly greater HHV-8 seropositivity (*p* = 0.009) and titers of HHV-8 antibodies (*p* = 0.005) than healthy controls. The seropositivity and blood titers of HHV-8 antibodies were similar in TB patients with and without effusions. Among TB patients with effusions, similar percentages had seropositive plasma and seropositive effusions. Plasma samples of 6 TB patients, but none of the healthy controls, were positive for HHV-8 DNA (*p* = 0.03). TB patients with or without effusions had lower blood lymphocyte counts and higher blood monocyte counts than healthy controls (*p* < 0.0001 for both). TB patients with effusions had significantly lower blood lymphocyte counts than those without effusions (*p* = 0.035).

**Conclusions:**

HHV-8 had similar seroprevalence in TB patients with and without effusions. However, TB patients with effusions had lower blood lymphocyte counts than those without effusions.

## Background

Previous studies have consistently identified human herpesvirus type 8 (HHV-8) DNA in patients with all types of Kaposi’s sarcoma (KS), and this virus is considered the etiological agent for KS [1, 2]. Healthy individuals with HHV-8 infections typically have no symptoms or signs of disease. However, HHV-8-infected patients with diverse immunologic abnormalities [3, 4], such as those with the human immunodeficiency virus (HIV) infection, can develop KS.

HIV-infected patients have a decreased number of circulating CD4+ T-lymphocytes. Depletion of T-lymphocytes in general and CD4+ cells in particular also occurs in patients with tuberculosis (TB) [5, 6]. Moreover, patients with HIV infection or pulmonary TB have peripheral blood mononuclear cells with impaired interferon-γ production upon in vitro antigenic stimulation, as well as aberrant in vivo T-cell activation [7]. There is also a high HHV-8 seroprevalence in HIV-infected or pulmonary TB patients without KS [8–11]. Primary effusion lymphoma (PEL) occurs mostly in HIV-infected patients, and is associated with HHV-8 infection [12]. The HHV-8 DNA levels in effusion samples from patients with PEL are significantly higher than those in blood samples [13]. However, the presence of HHV-8 antibodies and DNA in the plasma and effusions of TB patients with pleural or peritoneal effusion has not yet been determined.

Patients with active TB are more likely to have lymphopenia and monocytosis [7, 14, 15]. Thus, we evaluated the associations of HHV-8 infection with lymphopenia and monocytosis in TB patients with and without effusions. In particular, we assessed the positivity and titers of HHV-8 antibodies and the positivity and levels of HHV-8 DNA in blood and effusion samples of TB patients with pleural or peritoneal effusions.

## Methods

### Subjects and sample collection

All patients had newly diagnosed pulmonary TB and were examined before treatment. TB was initially suspected based on clinical features and abnormalities on chest radiographs, and confirmed by identifying *Mycobacterium tuberculosis* from sputum cultures in Löwenstein–Jensen medium. Patients with abdominal fullness, fever, and even dyspnea were suspected of having peritoneal effusion. These patients were given chest and/or abdominal CT exams, with aspiration of the peritoneal effusion using an echo-guide. The collected pleural and peritoneal effusions were studied for routine biochemistry including polymerase chain reaction (PCR) and mycobacterial culture for TB detection, and measurement of adenosine deaminase (ADA). Definite TB effusion was indicated by positive culture or PCR results. Patients without culture or PCR positivity were reassessed 2 months after the initiation of anti-TB treatment if they had a predominance of lymphocytes, high level of serum protein, and ADA greater than 40 mg/mL. Because of the high prevalence and the need for legal notification, the final TB diagnosis was discussed and determined by an official committee of Centers for Disease Control of Taiwan based on clinical information and treatment outcome. Patients with additional heart, lung, or other major diseases were excluded. The study protocol was approved by the Institutional Review Board of the Buddhist Dalin Tzu Chi Hospital (B09602001 and B09703021). All individuals provided informed written consent for participation.

Plasma samples were collected following medical examinations of 129 patients with pulmonary TB and 129 age- and sex-matched healthy controls. Forty of the TB patients had pleural or peritoneal effusions, and 38 of these effusions were available. All samples were collected in sterile tubes and centrifuged immediately at 4 °C to remove cells. Aliquots of the supernatants were frozen at −70 °C until analysis of HHV-8 antibodies and DNA.

The lymphocyte and monocyte counts of peripheral blood from healthy controls and TB patients were analyzed using an automated hematologic analyzer (XE-2100, Sysmex, Kobe, Japan) before plasma sample collection.

The mean ages of the 91 male controls (62.5 ± 12.6 years) and the 38 female controls (58.9 ± 17.2 years) were similar (*p* = 0.18; *t* test), as were the mean ages of the 91 male TB patients (62.3 ± 12.6 years) and the 38 female TB patients (58.9 ± 17.2 years) (*p* = 0.22; *t*-test). The mean ages of TB patients with effusions (60.1 ± 17.1 years; 29 men and 11 women) and without effusion (61.8 ± 12.6 years; 62 men and 27 women) were also similar (*p* = 0.52; *t*-test). All patients and controls were negative for HIV antibodies.

### Immunofluorescence assay (IFA) for HHV-8 antibodies

A commercially available IFA kit (Advanced Biotechnologies Inc, Columbia, MD) was used to detect HHV-8 immunoglobulin G (IgG) antibodies against the lytic antigens in plasma and effusion samples according to the manufacturer’s instructions. This assay was based on HHV-8-infected body cavity lymphoma cell lines. Plasma or effusion samples at various dilutions were brought into contact with fixed infected cells and examined microscopically using a fluorescence microscope. Samples that displayed fluorescence at a dilution of 1:40 were considered positive. Maximum HHV-8 antibody dilutions were determined by end-point IFA.

### DNA extraction and amplification of HHV-8 DNA

HHV-8 DNA was extracted and PCR-amplified from each patient’s plasma and effusion as reported previously [11].

### Chemiluminescence immunoassay for HIV antibody

Plasma samples were assayed for antibodies to HIV-1 and HIV-2 using the Vitros anti-HIV 1 + 2 reagent pack, control, and calibrator (Ortho-Clinical Diagnostics, High Wycombe, England) and the Vitros ECi immunodiagnostic system (Ortho-Clinical Diagnostics, Rochester, NY) according to the manufacturer’s instructions.

### Statistical analysis

A *χ*^2^ test or Fisher’s exact test was used to assess the significance of between-group differences in categorical variables. The significance of differences in the means of continuous variables from two groups was determined using the *t*-test. The comparison of plasma anti-HHV-8 titers in healthy controls and TB patients was analyzed using the Mann–Whitney test. A p-value less than 0.05 was considered significant. Statistical analyses were performed using SPSS version 12.0 for Windows (SPSS, Chicago, IL).

## Results

Twenty-two healthy controls (17.1 %, 17 males and 5 females) and 40 TB patients (31.0 %, 29 men and 11 women) were positive for HHV-8 antibodies. The HHV-8 seropositivity rate and titers of HHV-8 antibodies were significantly greater in TB patients than in healthy controls (*p* = 0.009 by *χ*^2^ test and *p* = 0.005 by Mann–Whitney test, respectively) (Table 1). The mean ages were similar in healthy subjects who were seropositive and seronegative, in TB patients who were seropositive and seronegative, and in TB patients with or without effusions (*p* > 0.05 for all 3 comparisons; *t*-test). The seropositivity rate was also similar in male and female healthy controls, in male and female TB patients, and in TB patients with and without effusions (*p* > 0.05 for all 3 comparisons by *χ*^2^ or Fisher’s exact test).

**Table 1:** Age, lymphocyte count, monocyte count, HHV-8 seropositivity, titer of anti-HHV-8 antibodies, and presence of HHV-8 DNA in TB patients and in age- and sex-matched healthy controls

	Age^a^ (years)	Lymphocyte count^a^/μL	Monocyte count^a^/μL	IFA (+) rate^b^	Anti-HHV-8 with maximal dilution						HHV-8 DNA
					1:40	1:80	1:160	1:320	1:640	1:1280	
Healthy controls	61.4 ± 14.1	1829 ± 495	315 ± 110	17.1 % (22/129)	15	6	0	0	1	0	0
*P* value		<0.0001^c^	<0.0001^c^	0.009^d^	0.005^e^						0.03^f^
TB patients	61.3 ± 14.1	1291 ± 661^g^	552 ± 291^g^	29.7 % (40/129)	21	11	2	2	3	1	6

*HHV-8* human herpesvirus type 8, *IFA* mmunofluorescence assay, *TB* tuberculosis

^a^Mean ± standard deviation. ^b^Positive in the IFA. ^c^ *t*-test. ^d^χ^2^ test. ^e^Mann–Whitney test. ^f^Fisher’s exact test. ^g^Lymphocyte and monocyte counts of 9 TB patients were unavailable

Patients with effusions (*n* = 40) and without effusions (*n* = 89) had similar HHV-8 seropositivity, titer of HHV-8 antibodies, and HHV-8 DNA positivity in plasma (*p* = 0.56 by *χ*^2^ test; *p* = 0.45 by Mann–Whitney test; and *p* = 0.67 by Fisher’s exact test, respectively) (Table 2).

**Table 2:** Age, lymphocyte count, monocyte count, HHV-8 seropostitivity, titer of anti-HHV-8 antibodies, and presence of HHV-8 DNA in TB patients with and without effusions

	n	Age^a^ (years)	Lymphocyte count^a^/μL	Monocyte count^a^/μL	IFA (+) rate^b^	Anti-HHV-8						HHV-8 DNA
						1:40	1:80	1:160	1:320	1:640	1:1280	
TB patients with effusion	40	60.1 ± 17.1	1112 ± 452	558 ± 290	27.5 % (11/40)	7	3	0	1	0	0	1
*P* value		0.52	0.035^c^	0.87^c^	0.56^d^	0.45^e^						0.67^f^
TB patients without effusion	89	61.8 ± 12.6	1380 ± 729^g^	549 ± 293^g^	32.6 % (29/89)	14	8	2	1	3	1	5

*HHV-8* human herpesvirus type 8, *IFA* mmunofluorescence assay, *TB* tuberculosis

^a^Mean ± standard deviation. ^b^Positive in the IFA. ^c^ *t*-test. ^d^χ^2^ test. ^e^Mann–Whitney test. ^f^Fisher’s exact test. ^g^Lymphocyte and monocyte counts of 9 TB patients without effusions were unavailable

The effusion samples of 2 TB patients with pleural effusions were unavailable, but the plasma of 1 of these patients was positive for HHV-8 DNA (472 copies/mL) (Table 2). The positive rates and titers of HHV-8 antibodies in the plasma and effusion samples of the remaining 38 patients with effusions (3 with ascites and 35 with pleural effusion) were similar (*p* = 0.45 by *χ*^2^ test; *p* = 0.50 by Mann–Whitney test, respectively) (Table 3). The plasma and effusion samples of the 38 patients whose effusions were available were all negative for HHV-8 DNA.

**Table 3:** Number of TB patients who tested positive for anti-HHV-8 antibodies in plasma and in peritoneal or pleural effusion samples

	Plasma	Effusion	*P* value
IFA + ^a^	10/38 (26.3 %)	13/38^b^(34.2 %)	0.45^c^
Anti-HHV-8 titers			0.50^d^
1:40	7	10	
1:80	3	3	

HHV-8, human herpesvirus type 8; IFA, immunofluorescence assay; TB, tuberculosis

^a^Positive in the IFA. ^b^Effusion specimens of 2 of the 40 TB patients with effusions were unavailable. ^c^χ^2^ test. ^d^Mann-Whitney test

The plasma samples of 3 of the 89 patients without effusions who were negative for HHV-8 antibodies were positive for HHV-8 DNA (544, 899, and 1011 copies/mL). Moreover, 2 of the patients without effusions who were positive for HHV-8 antibodies were positive for HHV-8 DNA (1415 and 3720 copies/mL). Thus, the plasma samples of 6 TB patients, but none of the healthy controls, were positive for HHV-8 DNA (*p* = 0.03) (Table 1).

TB patients had much lower blood lymphocyte and monocyte counts than healthy controls (*p* < 0.0001 for both; *t*-test) (Table 1). However, controls who were seronegative and seropositive, and patients who were seronegative and seropositive had similar blood lymphocyte counts (1838 ± 501/μL vs. 1783 ± 471/μL and 1322 ± 656/μL vs. 1222 ± 673/μL, respectively; *p* > 0.05 for both; *t*-test) and similar blood monocyte counts (318 ± 109/μL vs. 301 ± 115/μL and 546 ± 251/μL vs. 565 ± 368/μL, respectively; *p* > 0.05 for both; *t*-test).

TB patients with effusions had significantly lower blood lymphocyte counts than those without effusions (1112 ± 452/μL vs. 1380 ± 729/μL; *p* = 0.035; *t*-test) (Table 2). Among TB patients with and without effusions, blood lymphocyte counts were similar for those who were HHV-8 seronegative and seropositive (1125 ± 448/μL vs. 1078 ± 484/μL and 1428 ± 727/μL vs. 1283 ± 793/μL, respectively; *p* > 0.05 for both; *t*-test). Blood monocyte counts in HHV-8 seronegative and seropositive TB patients with and without effusion were also similar (534 ± 222/μL vs. 621 ± 428/μL and 552 ± 268/μL vs. 542 ± 346/μL, respectively; *p* > 0.05 for both; t-test).

None of the TB patients who were positive for HHV-8 antibodies or HHV-8 DNA had clinical manifestations of HHV-8 infection, such as KS, PEL, or Castleman disease.

## Discussion

The major finding of the present study is that HIV-negative individuals with pulmonary TB, both with and without effusions, had greater seropositivity for anti-HHV8 antibodies than age- and sex-matched healthy controls. These results are in line with the results of our previous study of subjects with TB pneumonia [11]. The cutoff point for HHV-8 seropositivity in the present study was set at 1:40 according to the manufacturer’s instructions. In our recent study of HHV-8 seroprevalence in patients with end-stage renal disease, one of the two healthy controls with IFA antibody titers of 1:160 displayed positivity in a specific enzyme-linked immunosorbent assay (ELISA) [16]. If the cutoff point for seropositivity was set at 1:160 in our previous study, then the seropositive rate among TB patients was marginally higher (5/101 vs. 0/101; *p* = 0.059; Fisher’s exact test) [11], but in the present study the seropositive rate would remain significantly higher in patients than in controls (8/129 vs. 1/129; *p* = 0.036; Fisher’s exact test). This discrepancy might be due to fewer subjects in the previous study.

The present study demonstrated that the seroprevalence of HHV-8 was similar among TB patients with and without effusions (27.5 vs. 32.6 %). As noted in our previous study of subjects with pulmonary TB [11], HHV-8 seroprevalence in HIV-negative TB patients with pleural effusions was lower than in HIV-negative patients with KS (52–100 %) and comparable to that in HIV-positive patients without KS (13–50 %) [8–10, 17]. This suggests that HHV-8 infection has similar roles in TB patients with and without effusions. However, the study groups enrolled in the present and previous studies were much older than individuals who have the greatest risk for HIV infection.

Previous studies reported that the prevalence of HHV-8 infection increases with age [18–20], and that KS is more common in elderly males [21]. Our previous comparison of TB patients and healthy controls [11] indicated similar prevalence of HHV-8 seropositivity for males and females and similar mean ages for seropositive and seronegative subjects. In agreement, the present study demonstrated similar seropositivity for males and females, similar mean ages for seronegative and seropositive subjects, and similar seropositivity for TB patients with and without effusions.

Our previous study reported similar positivity for HHV-8 DNA in TB patients and healthy controls (4/101 vs. 0/101, *p* = 0.12) [11]. However, the present study indicated that HHV-8 DNA positivity was significantly greater in the TB group than in the control group (6/129 vs. 0/129, *p* = 0.03). These discrepant findings might be attributable to smaller number of subjects in our previous study.

Our previous study of patients with cirrhosis and ascites indicated the positive rate and titers of anti-HHV-8 antibodies were much greater in plasma than ascites (*p* < 0.0001) [22]. Among cirrhosis patients, the positive rate of anti-HHV-8 antibodies in plasma samples was more than 6-fold greater than in effusion samples. By contrast, the present study indicated similar positive rates and titers of anti-HHV-8 antibodies in plasma and effusion samples among TB patients with effusions (13/38 vs. 10/38, *p* = 0.45). The most likely reason for this difference is that TB effusions are exudates, but cirrhosis effusions are transudates [23].

HHV-8 is associated with PEL, a rare form of malignant clonal B-cell lymphoproliferation [12]. Among patients with PEL, the level of HHV-8 DNA in effusion samples is significantly higher than in blood samples [13]. In contrast, the present study found that plasma and effusion samples from the 38 TB patients with available effusions were all negative for HHV-8 DNA. This suggests that the role of HHV-8 is different for patients with TB and those with PEL.

The present study found that TB patients with or without effusions had lower lymphocyte counts and higher monocyte counts than healthy controls (*p* < 0.0001 for both), in agreement with our previous study of patients with TB pneumonia [11]. Stimulated pleural macrophages can induce production of interleukin-8 (IL-8) protein, and IL-8 in turn can induce lymphocyte chemotaxis into the pleural space [24]. Hence, lymphocyte counts are elevated in the effusions of patients with TB [25]. This may explain the finding of the present study that TB patients with peritoneal or pleural effusions had significantly lower blood lymphocyte counts than those without effusions (*p* = 0.035).

These are four limitations in the present study. First, although this is the first study to comprehensively investigate the prevalence of HHV-8 in TB patients with peritoneal or pleural effusions, all participants were recruited from a single teaching hospital. Thus, the results may not be representative of the overall situation in Taiwan or elsewhere in the world. Second, the PCR method used in this study can only detect 5–10 copies of HHV-8 DNA, and therefore may be insufficiently sensitive for definitively establishing the presence or absence of HHV-8 DNA. The question of whether patients such as those who participated in this study have HHV-8 DNA at levels less than 5–10 copies/mL be resolved if the detection sensitivity can be increased. Third, we did not investigate the potential for a common underlying cause of both TB and HHV-8 infection, such as socioeconomic status, nutritional conditions, and health-related behaviors. Fourth, we did not have records of any co-morbidities or adjunct therapies in our TB population, and we expect these to be relatively common in older individuals, such as those in our study population.

## Conclusion

The seropositivity and titers of anti-HHV-8 antibodies and the seropositivity of HHV-8 DNA were significantly higher in TB patients with or without peritoneal or pleural effusions than in healthy controls. The prevalence of HHV-8 in TB patients with or without effusions is comparable to that previously reported for HIV-infected patients without KS. Thus, our study suggests that TB patients with or without effusions are vulnerable to HHV-8 infection, similar to HIV-infected patients. We also found that the seropositivity and titers of anti-HHV-8 antibodies and the seropositivity of HHV-8 DNA were similar for TB patients with and without effusions, suggesting a similar role for HHV-8 in these two groups of TB patients. TB patients with effusions had significantly lower blood lymphocyte counts than those without effusions. This may be attributable to IL-8 production mediated by stimulated peritoneal or pleural macrophages and the subsequent induction of lymphocyte chemotaxis into the body cavities.

## Abbreviations

ELISA: Enzyme-linked immunosorbent assay
HHV-8: Human herpesvirus type 8
HIV: Human immunodeficiency virus
IFA: Immunofluorescence assay
IL-8: Interleukin-8
KS: Kaposi’s sarcoma
ORF: Open reading frame
PCR: Polymerase chain reaction
PEL: Primary effusion lymphoma
TB: Tuberculosis.
